# A Practical Treatise on Amaurosis, Containing New Researches on the Particular Kinds of Treatment Adapted to Its Various Species

**Published:** 1844-01

**Authors:** 


### Art. IX.

*Traité Pratique de l'Amaurose ou Goutte-Sereine, avec des Recherches Nouvelles sur les Méthodes spéciales de Traitement qui conviennent à ses differentes Espèces.* Par J. E. Pétrequin, D.M.P.—*Paris*, 1841. 8vo, pp. 120.

*A Practical Treatise on Amaurosis, containing new Researches on the particular kinds of Treatment adapted to its various Species.* By J. E. Pétrequin, M.D.—*Paris*, 1841.

Along with much that is valuable, there is a good deal of flourish and pretence in the work now before us. The author talks perpetually of his new researches, new studies, new ideas, new therapeutical views. We have been able to detect almost nothing that is new. We defy M. Pétrequin to put his finger on any one fact, or any one opinion in his book, which is not to be found fully discussed in the writings of his predecessors. Our only reason for directing our readers' attention to M. Pétrequin's work is, that, if his cases are not exaggerated, he has been very successful in the treatment of amaurosis, and we consider it important to inquire to what this success has been owing. He tells us, that a radical cause of failure, in the practice of many, is the notion, that amaurosis is simply a palsy, and ought to be treated as such. His own success he attributes to an accurate diagnosis of the different species, and to a treatment appropriated to each, but varied according to their periods and complications. He ascribes much also to his establishment of the species, not according to the seat of the disease, which he thinks must always be problematical, but according to the essence or nature of the cause. This is a very reasonable view of the matter. To distinguish, for example, between an amaurosis affecting the retina and one seated in the optic nerve, must be frequently impossible, and even were the diagnosis made, it would generally be of no manner of use; but to confound a congestive amaurosis with one of an opposite kind might lead to the most serious evil.

The work is divided into two parts: the first consisting of particular observations, and the second of general reasonings.

The following is an analysis of the facts presented to our consideration in the first part.

*Asthenic nervous amaurosis.* M. Pétrequin tells us, that this species of amaurosis is the most simple, the least complex. When primary, it arises chiefly from an inordinate exercise of sight, or from some debilitating influence; such as want, age, narcotics, venery, hemorrhage, typhus, &c. It is often consecutive, originating in some other variety of the disease, as the verminous, the traumatic, or the congestive. Sometimes the cause is local, sometimes general.

The course of asthenic amaurosis is slow and free from pain. The pupils are dilated, and their motions impeded; the patient complains of visus reticulatus, and has a dull expression of countenance. This species often assumes the form of night-blindness. The patient searches for more light, and often changes his glasses in the hope of seeing better. The prognosis has generally been unfavorable, but to this M. Pétrequin cannot agree.

A tonic plan of treatment should be followed, both internally and externally, strengthening diet, the preparations of iron and of cinchona, frictions with the tincture of nux vomica, aromatic vapours, stimulating collyria, blisters to the forehead dressed with strychnia, and electricity. Setons in the neck, so much used, are hurtful in asthenic amaurosis, as well as exclusion of light from the eyes by shades.

CASE I. The left eye of a woman, aged twenty-five, employed in embroidering, is affected. No photopsia, no muscæ, no pain of head or eyes. Pupil a little dilated. Everything seems as if in a whitish cloud. A cure is effected by blisters and the application of strychnia and nux vomica; ammoniacal vesication; and frictions with tincture of nux vomica.

CASE II. The right eye of a tailor, aged twenty-four, becomes incompletely amaurotic, after bathing in the river. Headach, mistiness of vision, myopia, muscæ, with a little dilatation of the pupil. Cured by frictions with tincture of nux vomica.

CASE III. To intermittent fever there supervene anæmia, anasarca, and debility, with incomplete amaurosis, and dilated, but moveable, pupils. No pain nor photopsia. Cured by good food, strychnia and powdered nux vomica repeatedly to the blistered forehead, and frictions with tincture of nux vomica.

*Amaurosis from lightning.* In its symptoms and nature, M. Pétrequin regards this amaurosis as approaching the asthenic or torpid, and, therefore, as requiring the same treatment. He considers it as a result of a *perturbation of the nerves* and of a *subtraction of the nervous fluid,* phrases to which no accurate signification can be attached.

Case iv. The conductor of a diligence is deprived of sight by a stroke of lightning. He has been bled, leeched, had emetics and purgatives, and been blistered, without benefit. Cured after fifteen months, by the internal use of alum. Probably a natural cure.

Case v. A sailor is struck blind by lightning. His eyes appeared so natural, that he was suspected to be simulating. Various remedies were tried, but with no effect. Gradually he begins to see in the dark, then during day, and ultimately recovers perfectly, probably in consequence of careful regimen.

*Amaurosis from worms.* The disease of the eyes which arises from worms in the intestines is at first only a mydriasis, and may readily be cured by anthelmintics. If the case is neglected, the retina becomes implicated, and then the removal of the worms may prove of no avail. The amaurosis approaches the asthenic. The countenance is tumid, and of a pale yellowish hue; the belly distended; the head heavy; there is nausea, with itching of the nostrils and throat, disturbed sleep, and twitchings of the face. The anthelmintics chiefly recommended by M. Pétrequin are worm-seed (artemisia Judaica,) and Corsican wormweed (fucus helminthocorton.)

Case vi. A child is cured of complete amaurosis, after passing a knot of lumbrici.

Case vii. A girl of fourteen passes sixty lumbrici, and is cured of amaurosis. M. Pétrequin merely saw the worms at Padua.

Case viii. A girl, twelve years old, after having been troubled with worms, and suffering from tinea favosa, becomes incompletely amaurotic. No pain; pupils dilated, but contractile; complains of the sensation of a black mist; sees distant objects; diplopia. Passes no worms, but is cured by vermifuges, especially wormseed and Corsican wormweed. Appetite and general health improve under this plan, and patient gains flesh. It is not clear that the amaurosis arose from worms.

*Traumatic amaurosis.* M. Pétrequin refers traumatic amaurosis to three principal causes, viz. contusion of the eye, concussion of its nervous system, and injuries of its accessory nerves. It generally occurs instantaneously. Sometimes it yields as the symptoms by which it is accompanied subside under treatment, but often it persists, under an asthenic character. M. Pétrequin supposes an analogy to exist between the injuries of the eye which determine amaurosis, and concussion of the brain producing palsy. The pupils are dilated and irregular.

The *first* indication is to combat the general effects of the injury; the *second,* to attack the congestive or other complications; and the *third,* should the disease continue under an atonic form, to employ either the means already recommended for asthenic amaurosis, or those hereafter to be pointed out for the cure of the torpid variety.

Case ix. A fall on the head, from a considerable height, produces concussion of the brain, which is successfully treated by bloodletting and other remedies, but vision is double, and that of the right eye is short and indistinct. The right pupil was at first dilated; now the pupils are natural, and there are no signs of inflammation, congestion, nor irritation. The visual asthenia is treated by frictions with tincture of nux vomica. Next morning, no diplopia. The frictions are omitted, and double vision returns. They are resumed, vision again becomes single, and a complete cure is effected under a continuance of the remedy.

Case x. By an explosion in a mine, a strong man is thrown to a distance of fifteen paces. Amongst other consequences of the injuries received, the eyelids are œdematous, the conjunctivæ ecchymosed, and the patient deprived of sight. The cerebral symptoms are combated by bloodletting and other remedies. Fifteen days after the accident, the patient sees a little with the left eye, but the right continues completely amaurotic. Sixteen leeches behind the right ear restores the left eye nearly to a natural state, but the vision of the right eye is still defective. Pupils unequal in size. A gradual recovery takes place, without any particular treatment being mentioned.

Case xi. By an explosion in a mine, a man, among other injuries, receives two deep wounds on the forehead, and has his left eye destroyed entirely, while the right presents a superficial scratch of the cornea, with a dilated and irregular pupil, which is displaced upwards and motionless, and a loss of vision. The cerebral concussion and other symptoms are treated by bloodletting and abstinence. On the tenth day, vision of right eye begins to return, the pupil remaining dilated and irregular, but moveable. The patient complaining of weight in the head, fifteen leeches are applied behind the right ear. Vision improves, but it is still dim and easily fatigued. Six weeks after the injury, the visual asthenia continuing, frictions with tincture of nux vomica are had recourse to, and in three days a great improvement takes place. In six days, the sight of the right eye is perfectly restored.

Case xii. A fall on the right cheek is followed by epistaxis and insensibility, and by ptosis and complete amaurosis on the right side. Pupil dilated and motionless; malar bone depressed; palsy of the zygomatici, dilatator nasi, buccinator, and masseter; deafness of right ear; loss of speech; lower jaw motionless. Fifteen leeches are repeatedly applied behind the right ear. Sensibility returns, but the patient can neither speak nor chew. The blindness and deafness continue. Skin round orbit becomes ecchymosed. Leeches are again employed, and on the sixth day, the muscles of the lower jaw resume their office, and the ptosis disappears. On the seventh day, the patient begins to see a little, and the other symptoms gradually abate, till the twenty-third day; when, being exposed to the air and to the sun, the patient is seized with headach and other alarming symptoms; sixteen leeches relieve him, the pupil becomes normal, and vision is restored.

*Congestive amaurosis.* This depends on vascular derangement of the organ of vision. M. Pétrequin distinguishes three principal varieties of it, which he designates by the names of *simple congestive, irritative sanguineous*, and *irritative nervous* or *erethistic*.*

He has seen *simple congestive amaurosis* arise, especially in some debilitated individuals, without any premonitory symptoms, without any

* From ἐρέθω, I irritate, provoke, disturb.

ulterior signs of irritation, and without photophobia. He has seen it attack the eye suddenly, like an apoplexy, or commence with myopia, and manifest itself with dilated pupils, as an atonic congestion. It bears considerable resemblance to asthenic amaurosis, from which it is distinguished by its vascular origin, the circumorbital feeling of weight which attends it, &c. In cases where sanguineous evacuations would have weakened the patient too much, M. Pétrequin has used cutaneous revulsives and laxatives, with good effect.

The *sanguineous*, or *hyperæmic irritative amaurosis* is the most common, and is generally met with in subjects about the prime of life and of a plethoric constitution, after some violent excitement of the brain or of the eyes. It depends on an active congestion of the organ of vision, either showing itself at once, or developed by degrees. It is much influenced by any disorder of the cerebral circulation, and is generally attended by evident signs of irritation, as headach, sudden confusion of sight, photopsia, photophobia, vertigo, &c. At the long run, it is not confined to a merely congestive form, but attacks the eye still more seriously, and leads to a chronic sub-inflammation of the retina. The sclerotic becomes injected; the pupil contracted and irregular. The patient complains of muscæ, and of pain deep in the orbit and in the temple. He has the sensation of fire flashing in his eyes. If the flashes change from white to blue, violet, yellow, or red, the disease is increasing; if from red or yellow to violet, blue, or white, it is subsiding.

Except in cases of excessive plethora, M. Pétrequin does not bleed much from the arm. He prefers leeches, placed round the anus, so as to act on the portal system and the liver; afterwards he plants them behind the ears, so as to affect more directly the congested organs; the congestion being relieved by leeching, he blisters the nape. Each application of leeches, he follows up with a saline purge, and afterwards keeps the belly open with pills composed of calomel and aloes, sometimes adding to them extract of belladonna. He causes the forehead to be rubbed with blue ointment, mixed with belladonna. He speaks of mercury in high terms of eulogy; as removing, if properly administered, obstructions and irritations of the retina.

The *nervous irritative*, or *erethistic*, *amaurosis* is met with chiefly in females, and nervous subjects, who have suffered from neuralgia, or depressing passions, or whose brains have been over-excited by intellectual labours. Contraction of the pupil, over-sensibility of the retina, chroopsia, an improvement in vision by twilight or moonlight, myodesopsia, and lacrymation are among the local symptoms. At first, objects appear too much illuminated; then they seem enveloped in a vaporous veil, which thickens more and more. Externally, anodynes are to be used to the eyes, as a collyrium of lettuce-water or of laurel-water, with an addition of the aqueous extract of opium; frictions of the forehead with hydriodate of potass ointment are to be employed; and the warm bath. Internally, Méglin's pills* are recommended; also, extract of hemlock, cyanide of potassium, prussic acid, laurel-water, inspissated juice of lactuca sativa, antispasmodics, ether, emollient enemeta, asses' milk, sometimes chalybeates, certain mineral waters, &c.

* Méglin's pills consist of the extracts of valerian, fumatory, and hyoscyamus, with oxide of zinc, of each 1 oz., divided into 576 pills.

Both in the sanguineous and in the nervous irritative amaurosis, coloured glasses are to be used, moderate exercise, abstinence from fatiguing occupations, pediluvia with mustard, laxatives, a mild vegetable diet, cold fomentations of the forehead and eyelids, amusing employments, &c.

Case xiii. Fifteen months after being cured of a catarrhal ophthalmia, a shoemaker, of lymphatico-sanguine temperament, is suddenly affected with incomplete amaurosis, without weight or pain in the head. Every thing appears as if through a yellowish cloud; no photopsia; pupils moveable. Cured by fifteen leeches to the anus, low diet, pediluvia with mustard, and laxatives.

Case xiv. A hatter, weak-sighted for three years, becomes amblyopic. Cannot see to walk the streets; grayish mist before him; amaurotic expression of face, occasional giddiness; no pain, sleep disturbed. Recovers after venesection, purgatives, and frictions of the forehead with blue ointment.

Case xv. A young woman, aged seventeen, of sanguine constitution, loses the sight of the right eye, with pain in the orbit, photophobia, and myodesopsia; pupil dilated; cannot see her fingers. The symptoms yield considerably to venesection, three applications of ten leeches, and two purges. After a blister to the nape, deciphers letters half an inch long, but the sight is still short and indistinct. A perfect cure is speedily effected by frictions of the forehead with tincture of nux vomica. M. Pétrequin congratulates himself on his tact in seizing the moment for changing the anti-congestive to the anti-asthenic means of cure.

Case xvi. A journeyman, aged eighteen, of robust constitution, drinks white wine, so as to make himself drunk for three days successively, in order to cure himself of ague, under which he has been labouring for three months. The fever leaves him at the end of fifteen days, but he is seized with weight in his head, and intense pain in the forehead and hindhead, followed by muscæ, and rapidly increasing deterioration of vision. A year after the commencement of these symptoms, he is treated by M. Bonnet with bloodlettings, blisters, and strychnia, but without effect.

He comes under the care of M. Pétrequin, who finds the pupils moderately dilated, regular, and slightly contractile, the patient still complaining of headach, and pain deep in the orbits, able to count his fingers at the distance of six inches, but seeing nothing beyond a few paces, complaining of a dark mist and black muscæ, and presenting the stupor and expression of an amaurotic. Is bled, has fifteen leeches behind one ear, and fourteen behind the other, all in the course of four days, by which means the pain of the head is diminished. Twenty leeches are placed round the anus. Head is now heavy rather than pained; dartings in temples. After twelve leeches behind each ear, and a blister to the nape, there is no headach, pupils less dilated, and although the patient will not admit there is any change in his sight, it is plain, from the manner of his walking about, that he sees more than he did. Frictions on the forehead with blue ointment, mixed with belladonna, being employed, and a second blister to the nape, pupils become natural and contractile, and vision improves, so that he distinguishes objects at fifteen paces distance. Ammoniacal blisters are applied above right eye, dressed with one sixth of a grain, then one quarter of a grain of strychnia, and two grains of powdered nux vomica. The pupils become very contractile, and the patient reads large letters. Continues to improve under frictions of the forehead with tincture of nux vomica.

Case xvii. A journeyman, aged twenty-one, begins to lose his sight. with headach, mistiness of vision, muscæ, and giddiness as if intoxicated, The last symptom subsides after venesection, but sight continues to fail. Pupils contracted, irregular, moveable; sight short and dim, pain in hindhead, sensation of a brown veil, and of black whirling muscæ, cannot distinguish colours, impression of light painful, lacrymation. At ten paces, cannot distinguish one person from another; brought near enough, can decypher letters three or four lines long, but they become speedily obscure. Pulse full and strong, skin hot.

Twenty-five leeches round the anus, a purge, belladonna dropped in a fluid form upon the eyes. Pupils do not dilate, but become very irregular. After fifteen leeches behind right ear, right eye improves. Twelve are placed behind left, and the patient purged, after which vision becomes clearer and more extended. Headach ceases, impression from light less painful, at seven paces distance distinguishes numbers two inches long. Frictions on the forehead with a mixture of two drachms of blue ointment and half a drachm of extract of belladonna; a blister to the nape. In a few days, begins to see houses at 500 paces distance, and reads some lines in a printed book. The mistiness becomes whiteish and semitransparent; pupils still contracted, but regular. Progressive improvement. A slight relapse, during which the mistiness again becomes blackish, and presents the appearance of a wheel going round. Another blister to the neck, after which the mistiness disappears. Vision becomes clear, and of natural extent. Reads with ease. The treatment extended to about six weeks.

Case xviii. A woman, aged thirty-four, of lymphatico-sanguine constitution, menstruating regularly, is seized with pain in the temples, and awakes one morning with diplopia and indistinctness of sight. Is bled, blistered, and vomited; disease makes progress; pupils contracted and irregular; axes of eyes not perfectly parallel; impression of light painful; sensation of clouds, coloured areolæ round objects, and red sparks; cannot see small objects; distinguishes a finger at five paces, beyond which sees the hand only, which disappears at ten paces. Temporal cephalalgia, more severe on left side; aspect of stupor.

Twenty leeches to the anus; next day, Seidlitz water; momentary appearance of the menses, five days before their regular period, ascribed to the action of the leeches; headach, pain in loins, diplopia; after three drachms of cream of tartar in veal soup, pain of loins gives way; pupils more regular and less contracted; less pain in forehead; blister to nape; headach is removed; left pupil normal, right dilated; frictions on forehead with blue ointment and belladonna; two doses of Seidlitz water. Much improved; sees objects single within and beyond ten paces; left eye well, right a little indistinct; no headach. Both eyes being open, sees double at ten paces, single within and beyond this distance. Frictions with tincture of nux vomica; pupils equal in size; sees well; no diplopia, except on looking much to the right. Treatment extended to twenty-five days.

Case xix. A labourer, at the age of twenty-five, was suddenly struck with amaurosis of both eyes, which began to yield in three weeks. Next year, he had another attack, which lasted two months. Each year afterwards, for four successive years, he had a relapse, affecting the eyes alternately. On the 10th of August, 1837, the right eye is struck blind in a moment; then the left in November, when the darkness of the right abated a little. After being bled, blistered, purged, and having fifty leeches behind the ears, here is a slight amendment. Four doses of tartar emetic reduced the right eye to its former state of blindness.

On the 1st of January, 1838, comes under the care of M. Pétrequin. Eyes clear, pupils moderately dilated, and contractile; both eyes amaurotic, the right completely so; myodesopsia, thick dark mist; with right eye does not see a person at two paces distance; with left, cannot distinguish characters two inches long; cannot see to read; constitution robust; temperament sanguine; no pain of head. Purgatives and small blisters were used, without amelioration. After two ammoniacal vesications above right eye, dressed with a quarter of a grain of strychnia, and three grains of powdered nux vomica, an improvement takes place, so that he reads with that eye. On the morning of 16th of January, can read large characters, at the distance of seven feet, with right eye. At 4 p.m., loses the sight of it suddenly; cannot see even a lighted candle with it; with left reads an ordinary type. Low diet, laxatives, four grains of quina. On the 19th, is bled; vesication above right eye, and strychnia repeated; two calomel pills; quina stopped. Next day begins to discern his fingers. On the 24th, amaurosis of left eye becomes suddenly more intense; cannot distinguish letters with it of less than an inch in size. A fourth vesication, above right eye. On the 29th, venesection; fifth vesication, above left eye. 1st February, calomel stopped, in fear of salivation. Aloes pills; frictions of forehead with tincture of nux vomica. 13th, sixth vesication above right eye. Improvement continues; reads letters half an inch long with left eye; right distinguishes a pen, &c. 18th, Seventh vesication. 22d, Return of muscæ; fifteen leeches behind right ear. Eighth vesication; vision more distinct. 25th, More muscæ; fifteen leeches behind right ear. 3d of March, Ninth vesication; left eye improves. 12th, Reads an ordinary type with it. 14th, Sees people 500 paces distant. Right eye so defective, that cannot distinguish the hand with it at a foot distance. It is submitted to the action of a galvanic battery of sixteen plates; at the end of an hour sees the hand, fingers, and nails with it; vision of left more clear and extensive. Patient is enchanted; but by the evening the amendment subsides; and next day, disappointed at having lost what he had gained by the galvanism, and terrified at a power which had done more for his sight in an hour than all the other means in months, he quits the hospital.

Case xx. A countryman, aged seventy, sanguine and plethoric, is seized in September 1837, with prolonged giddiness, while thrashing corn, followed by headach and impaired vision; all objects appear red; red and yellow muscæ; reddish mist; impression of light painful. In November, sight better after sunset: progressive loss of sight, especially in right eye. Is bled, purged, and blistered, without effect.

On coming under M. Pétrequin's care, 15th of June, 1838, right pupil dilated and fixed; pain in orbits, forehead, temples, and occiput, especially on the right side ; complete blindness of righ eye, which is to the patient as if covered by a thick red veil ; with left eye gets a glimpse of his fingers, but does not distinguish colours, nor see a person at some paces distance; red and yellow muscæ; in the evening, sees a little better; impression of light painful; amaurotic expression and stupor. After being bled, having twenty-five leeches round anus, and taking Seidlitz water, there is a marked amelioration in his sight; pains in head cease; sees well with left eye. Having imprudently looked for a long time at bright objects in broad daylight, and even at the sun, a relapse, with headach; left pupil becomes immoveable like right; pains in left leg. Twenty-five leeches being applied to the anus, and twelve behind right ear, and the patient purged with sulphate of soda, a renewed improvement takes place. Left eye progresses steadily ; right begins to discern objects; pain in right side of head is relieved by twelve leeches to the temple, and a purge. Left eye sees almost as well as before the commencement of the disease ; right still sees red ; blister to nape, and behind right ear; weight of head, with general heat and pricking of skin coming on, twenty-five leeches to anus, and this not giving permanent relief, venesection. Eyes continue to improve; frictions of head with belladonnized blue ointment. An attack of giddiness, headach, and disturbed vision, of apoplectic character ; relieved by venesection, mustard pediluvia, and laxative enemata. Sight of left eye being very good, and the right capable of distinguishing the fingers at some paces distance, the patient leaves the hospital.

Case xxi is, we think, incorrectly placed among the instances of congestive amaurosis. It is one of obscurity of vision resulting from ophthalmia, in one eye at least attended by opacity of the cornea, with contracted pupil, and synechia posterior.

*Torpid amaurosis.* Torpid or paralytic amaurosis is more frequently consecutive to one of the other varieties than primitive, although symptoms of torpor of the retina may sometimes declare themselves suddenly. It is of importance, to study the different sources of torpid amaurosis. M. Pétrequin has seen it arise from occult syphilis, and Weller from scrofula. From the cases related, we suspect the constitution of the patient has considerably greater influence with our author in determining the case to be torpid, than the local symptoms. All the cases he relates occur in lymphatic, scrofulous, or feeble subjects.

The causes of torpid amaurosis being numerous and varied, so also are the symptoms. Even from the commencement of the disease, it requires a vivid light to produce an impression ; there is consequently a sort of night-blindness ; then a dull veil seems to cover all objects ; the patient walks with his head elevated, as if to search for light ; he has a constant craving for a clearer illumination of objects. The eye loses its vivacity of expression and of movement ; the look becomes dull and amaurotic ; often the countenance becomes pale, as if blanched. The pupils are generally motionless, and also dilated, except when the original cause is a very irritative congestion, for this causes contraction of the pupils.

In the treatment, the morbid complications must first be removed; then the palsy of the retina must be attacked by strychnia, and the like.

Now, here we are, with a vast pretence to originality and discovery, landed just at the old point. Here we have *palsy* of the retina, and *torpor* of the retina, expressions which convey no other meaning than just this, that the sensorial power is lost; and instead of a fine and accurate diagnosis, here we have blindness from syphilis, blindness from scrofula, and blindness from various other causes, all huddled together, and confounded under the unmeaning name of *torpid amaurosis*

Case xxii is one of scrofulous ophthalmia, with opacities of the cornea, in which venesections, an opiate collyrium, purgatives, calomel, blisters, leeches, frictions with belladonnized blue ointment, and with tincture of nux vomica, afford some relief. We protest against such a case being arranged among the amauroses.

Case xxiii appears also to be one of ophthalmia, terminating in obscurity of vision. The pupils are irregular and immoveable; on the left crystalline there is a radiated spot; intermittent headach; amaurotic look; the patient does not see to guide herself, and does not discern even the place of the window, unless the sun be shining on it. Sight has been declining for twelve years. After the use of calomel, so as to affect the mouth; blisters; frictions of the forehead with belladonnized blue ointment; purgatives; and leeches behind one of the ears; considerable improvement takes place.

Case xxiv. A weaver, aged nineteen, scrofulous but robust, has, in three weeks, become completely amaurotic, after acute pain in the forehead and temples. Pupils dilated and motionless; does not distinguish night from day; constant nictitation; dull pain in the orbits, sharp in the forehead and temples, and occasionally in the occiput. After being bled, purged, and taking twelve grains of calomel, headach diminishes, and he begins to get a glimpse of the light. Twelve leeches are applied behind the ears; a little more perception of light. The pains of the head continuing, twenty leeches are applied to the anus, followed by Seidlitz water, and a blister to the nape. Headach removed; vision slightly improved. The patient is now seized with paraplegia, but vision continues to improve. After frictions of the forehead with tincture of nux vomica, pupils less dilated and more contractile. Strychnia is given internally in doses of one eighth of a grain. Vision improves so much that he can count the lines of a printed book, and make out the large letters. Gangrenous sores form over the sacrum and haunches, and lead to a fatal termination. On dissection, a portion of the spinal marrow is found of a brownish gray colour, and softened, a consequence of inflammation. Nothing morbid is detected in the optic nerves, nor brain. The retinæ and the rest of the eyeballs seemed sound, and the limbus luteus distinct, only in one of the retinæ there is congestion of the central artery.

Case xxv. A woman, aged thirty-six, affected at twenty with a long-continued hemorrhoidal discharge, at twenty-eight with flooding after parturition, and at thirty-two with a pustular eruption, for which she was twenty-five months under treatment, had for ten years been troubled with transient cloudiness of sight. On the 15th of March, 1838, she was struck with complete amaurosis of right eye. The left eye, which had never been good, seemed also going. Salivated from mercurial frictions. 6th of April, Both eyes completely amaurotic; cannot distinguish day from night; pupils dilated and fixed; bottom of eyes of a grayish black; no headach; mouth sore; redness and pain of throat, for which fifteen leeches are applied to neck, followed by a slight improvement. 18th, Has a glimpse of daylight, without distinguishing anything; facies amaurotica, pupils as before. Strychnia is commenced; an ammoniacal vesication is performed every four days, and the strychnia applied, till the 19th of May. Sight recovers, so that she sees small objects, and distinguishes letters nine lines long. Pupils continue dilated, but moveable.

Case xxvi. A waiter, aged twenty-four, of feeble constitution, discovered one morning on getting up, that he had lost the sight of his left eye, without headach, and without any other premonitory symptom than some degree of giddiness. He had formerly been at country work, and subject, in summer, to epistaxis, which had left him since changing his employment. This was in the end of 1837. Different means were tried, and after six months his sight gradually returned. He continued well till 12th of September, 1838, when he rapidly became deprived of the sight of both eyes, after headach, without giddiness. Pupils dilated and motionless; amaurotic expression; with difficulty distinguishes day from night. A black fog seems spread out before him; no photopsia; some pain in forehead and temples; appetite and digestion natural.

His feeble constitution forbade bleeding. After being twice purged with Seidlitz water, begins with left eye to perceive a lighted candle, like a little star; right eye distinguishes the hand at some inches distance, and a person at some paces, like a shadow. After a blister to the nape, and frictions on the forehead with blue ointment and belladonna, the headach disappears; pupils less dilated and more contractile; black fog diminishes; with right eye begins to count the fingers, and distinguish colours. In four days more, deciphers large letters, and gets a glimpse of houses 500 paces distant; with left eye, sees better to one side than straight forwards; a lighted candle seems red to it. In other four days, and in sunshine, distinguishes houses 500 paces distant with left eye; and with right, sees the windows of those houses, and reads letters two lines long. The fog threatening to return, the nape is blistered, and Seidlitz water prescribed. This is followed by an amelioration, but as there is some return of headach, twelve leeches are applied to the anus, followed by a purge; marked relief. For a time, patient suffers several relapses, and sometimes sees double. He is again blistered on the nape, returns to the frictions with blue ointment, and afterwards uses those with tincture of nux vomica. Sight improves; less fogginess; pupils regular. The right eye sees almost as well as before the disease; reads an ordinary type with it; with left deciphers letters three or four lines long, but they seemed veiled by a central semi-transparent mist; diet is improved; forehead blistered, and dressed with nux vomica. Occasional headach; reddish sparks; giddiness; fourteen leeches to the anus, with Seidlitz water next day, after which all the symptoms abate; judging the congestion to be passive, cinchona is now given, under which the general health improves; sight of right eye becomes very good; that of left extensive, and pretty clear, except in the centre. No more diplopia; reads fluently; feels well; appetite good; no headach.

Case xxvii. A young man, aged twenty-six, becomes blind, and at the same time palsied in his lower extremities. Confesses he had syphilis, but did not undergo an appropriate treatment. The amaurosis and palsy are attributed to the action of the syphilitic virus on some part of the head and spinal column. He is put on Van Swieten's liquor. In fifteen days, begins to see, and, after two months' anti-venereal treatment, is dismissed cured.

*Organic amaurosis.* Under this head M. Pétrequin comprehends those cases which result from an anatomical alteration of the constituent, or of the accessory parts of the organ of vision. Their characters are not uniform, but vary as much as do their causes. Thus, still less than of torpid amaurosis, can any general history be traced of the organic.

Case xxviii. A husbandman, aged twenty-four, is affected with exophthalmos on the left side, and complete amaurosis of the protruded eye. Intra-orbital pain, photopsia, diplopia, epiphora, a feeling of pressure in the orbit. The eyelids cannot be completely closed; the pain increases. In about a month the protrusion and pain diminish, an improvement attributed by the patient to the application of a blister to the arm, but the vision continues as before.

On coming under M. Pétrequin's care, the left eye is found to be pushed downwards, and to protrude two or three lines beyond its fellow; eyelids red, and slightly œdematous; motions of eye unimpeded; it distinguishes light from darkness; pupil dilated; patient cannot open the eye to more than half the natural extent. The treatment consists in the frequent application of leeches, behind and before the ear; purgatives; calomel; a blister to the nape. Vision speedily begins to improve, a progressive diminution of the exophthalmos takes place, the diplopia disappears. At his dismissal, he knows a pair of scissors at five or six feet distance, and distinguishes one person from another at eight or ten paces. M. Pétrequin regarded the symptoms as owing to inflammation of the fibrous and cellular structures within the orbit, and to traction of the optic nerve.

Case xxix. A carrier, aged thirty-seven, is affected with inflammation of the pharynx for thirteen months, accompanied by frequent vomiting. His eyes are red, and as if pushed from their sockets by the constant efforts to vomit. Sight very weak; emaciation. The affection of throat is cured by the application of alum. Eyes less prominent and less red; sight restored. Seven months afterwards, the affection of the throat recurs, with incomplete deafness of the left ear, and amaurosis of the left eye; pupil contracted and motionless; globe of the eye deformed and flattened on its lower side, with some degree of atrophy; no vision in left eye; pain in left temple. It is treated as before; vision soon begins to improve, as well as hearing; pupil continues contracted; sees objects pretty well. Smart pain supervening towards the ear, a blister is applied to the nape with relief; hearing restored.

Case xxx. A woman, aged forty-five, strong and of sanguine temperament, is seized with apoplexy, consequent to continued pain in the head; is insensible for two days, but comes to herself after being bled; is palsied on left side; both eyes amblyopic; pupils contracted, irregular and but slightly moveable; sight extensive, but for near objects not sufficiently clear to enable her to guide herself surely; the right eye is the better; sees chiefly to one side; with an effort deciphers letters three or four lines long; muscæ; photopsia; intelligence somewhat affected.

Sixteen leeches to the anus; purge; blister to the nape; smart pains in the palsied parts; brilliant photopsia. Twenty leeches to the anus; seidlitz water. Feels relieved; vision clearer. Headach; vivid photopsia during night. Blister to the right arm; laxative. Sight clearer, scarcely any muscæ. Headach returns. Leeches to anus; eight grains of calomel. Begins to read the numbers on the beds. Frictions on the forehead, with belladonnized blue ointment. Photopsia and muscæ fade away. General improvement. Pains of head rare. Friction of the paralytic limbs with camphorated oil. Sight quite restored, except that left eye does not see outwards. Return of headach to a slight extent; Some photopsia. Seidlitz water; friction of limbs with a mixture of camphorated oil and and tincture of nux vomica. Headach ceases; strength improves. Headach and photopsia return; general uneasiness. Leeches to anus, and laxatives prove beneficial. More headach. Ten leeches behind left ear give relief; new pains. Ten leeches behind right ear. Gradual improvement. Sight good; no muscæ, nor photopsia; begins to walk.

Case xxxi. A steam-boat fireman, at the age of twenty-two, had gonorrhœa and a chancre, followed by ulceration and perforation of the palate, eleven months after which he became affected with constant headach, grew pale, and found his ideas confused and memory short. Was bled and had his scalp rubbed with tartar emetic ointment. After a fit of drowsiness, wakes one morning with the left eye amaurotic, but as the right eye is good, quits the hospital, to attend his employment. Some days after this, perceives sight of right eye to be growing dim. A blackish mist seems to arise at the external angle of the eye, and advancing by degrees, in fifteen days crosses the visual axis, and approaches the internal angle. Headach increases. Intelligence enfeebled; memory confused; an expression of deep sadness; complains much of ennui; constant pain in the superior-posterior part of the head; great propensity to sleep. Left eye is better; at three feet distance, distinguishes with difficulty the number of fingers held up before him; pupil two lines in diameter, contracts slightly; eyelids œdematous; bright light fatiguing. Right eye more open than left; pupil more dilated; scarcely distinguishes light and shadow, if the object is placed in the direction of the axis of the eye, but if placed to the inner side of the axis, distinguishes it in part, or entirely, according to its size. This perception is very confused even at the distance of a foot; outwards or in front does not see a lighted candle.

The following treatment extended through the space of a month. Twenty leeches to the anus. Seidlitz water. Less pain of head. Less confusion in the sight of left eye; right begins to have a glimpse of objects in the direction of the visual axis; pupils less dilated. Distinguishes with right eye a pen at the distance of a foot. Improvement of right eye continues; distinguishes a pen five feet distant; left eye improves more slowly; cannot distinguish with it figures two inches high and twelve feet distant. Twelve leeches to the anus; two aloes pills. Right eye nearly equal to left; distinguishes letters two lines long; cannot yet read. Purged by aloes and calomel; blister to nape. Sees better with right than left eye; if he employs both eyes, vision less distinct; walks in the wards, and has lost almost entirely the amaurotic expression. Aloes continued, eight leeches before left ear. Continues to see a whitish fog before left eye. Dose of sulphate of soda. Amelioration continues, but makes no progress. With right eye reads, but not fluently, words in characters two lines long; with left merely makes out the letters. An epileptic fit. Stupor; slowness of conception; memory weak; somnolency. No change in vision. Aloes pills continued. Seidlitz water. Less expression of torpor. Sight good. Ten months afterwards returns with confirmed dropsy, idiocy, and amaurosis far advanced.

Case xxxii we deem it unnecessary to quote. It is one of torpid amaurosis, which resists a variety of treatment. Such also is Case xxxiii. They seem to be placed under the head of organic amaurosis by mistake.

Case xxxiv. A man of strong constitution, aged fifty-one, is completely amaurotic for six years; the eyes preserve their natural brilliancy; pupils dilated; fungus of right maxillary sinus, the walls of which are destroyed; the whole scalp is painful. The patient dies after being three months in the hospital. On dissection, the retina presents numerous folds, crossing each other in different directions, and not arranged in the common radiated way. The texture does not appear changed; only that besides the spot of Soemmerring, which is very distinct, two other spots are observed, somewhat larger than it, separate from one another, and of a brick colour. The neurilema is very thick, and forms a sheath, not completely filled by the nerve, which is considerably shrunk. As it leaves the eyeball, the nerve is less consistent than natural, so much so that before reaching the commissure, it consists of pulp only, and even that presents a manifest solution of continuity. The commissure is in a state of commencing atrophy, and at its anterior part has lost in some measure, the appearance of cerebral substance.

Such is a condensed view of the first part of M. Pétrequin's work, and will afford our readers a tolerably fair idea of the practical and highly important facts which he has recorded. The second part consists of his general views on the subject.

He first of all explains the *principle of his classification*, viz., that the amauroses must be grouped, not according to their seat, but according to their nature. This, no doubt, would be strictly correct and philosophical, were it not a fact that it is very often just as difficult to detect the nature or essence of the pathological state which has produced blindness, as it is to fix on the part or parts of the optic apparatus in which the morbid change resides. We shall mention two causes of the difficulty of detecting the nature of an amaurotic affection. The one is, that in some subjects the local disorder is completely at variance with the general or constitutional state of the patient. Congestive amaurosis, for example, may attack a debilitated subject. A second cause is the transition of one morbid state into another; the inflammatory or congestive, for instance, passing into the torpid or atrophic. By these remarks, we do not mean to controvert the statement of M. Pétrequin, but only to show the difficulty there is of carrying out his principle in practice, and the invincible necessity, therefore of following an empirical, not a rational, mode of treatment in many cases of the disease in question.

M. Pétrequin next makes some remarks on what he terms *the elements of the amauroses.* It is not the powerless state of the nerve of vision which is to be treated, but the sanguineo-congested condition of the optic apparatus in some cases, its anæmic state in others; it is the sub-inflammatory irritation of the optic nerve, in one instance, its participation in some general dyscrasia, in another, against which our remedies are to be directed. These, and such like elements, as M. Pétrequin chooses to call them, being overcome, if visual asthenia remains, it becomes the object of treatment.

The *transformations of the amauroses* is the next topic on which M. Pétrequin touches, observing that not only do the amauroses differ from one another, but that even the same amaurosis presents at different periods of its course marked differences in its form and nature. He notices particularly two transformations. The first regards the seat of the disease; for, as it is either on the increase or on the decline, amaurosis may affect more or less extensively the retina and the rest of the optic apparatus. The second regards its nature. Asthenic amaurosis, for example, is rarely primary, but supervenes as a degeneration of the traumatic. The nervous erethistic is transformed into the sanguineous irritative, and this again into the sub-inflammatory. In directing the treatment, the practitioner must bear in view such transitions. M. Pétrequin takes little account seemingly of the difficulty of tracing such transitions. He speaks of them without hesitation as things which can be positively determined, and the treatment changed accordingly.

M. Pétrequin is inclined to believe in an *amaurotic myopia.* He supposes that as the visual faculty becomes impaired, the sphere of its activity is lessened; or, in other words, that the patient, from an affection of the retina, becomes myopic, with no diminution in the clearness of sight. More frequently, however, this amaurotic myopia is attended with a certain obscurity of vision. Those who are affected in this way, not knowing that they labour under anything more than a shortness of sight, endeavour to relieve themselves by concave glasses of increasing depth. M. Pétrequin has also known some who changed their apartments, fancying the obscurity to be from without, not from within. He remarks, that the phenomena which appear at the outset of the greater number of the amauroses recur on their decline; myopia succeeds amaurosis, as well as precedes it. Hence it is, that the eye does not resume its natural vivacity immediately after the cure of the amaurosis is accomplished; the vision retains a peculiar indecision for a long time; and the physiognomy a sort of stupor, and a characteristic attitude, which is termed the *facies amaurotica.*

The myopia in question, M. Pétrequin believes to depend, not on any change in the refractive media of the eye, but solely on a vital cause, and he compares it to those morbid states of the other senses, and especially of the ear, in which it is necessary to bring the object much nearer to the organ, than in the healthy condition, to produce the usual impression.

With regard to the alleged frequency of *relapses in amaurosis*, M. Pétrequin remarks, that one cause is to be found in the nature of the disease, and that the original source of the amaurosis may predispose it more or less to relapse, according as it is asthenic, congestive, erethistic, torpid, or organic. A second cause consists in the perpetual exposure of the organ of vision to external stimuli, which should be avoided by moderating the light, not by excluding it entirely. For this purpose, coloured convex glasses of long focus are recommended by our author, and a long convalescence, before the eyes are seriously set to work. The patient should confine himself to *seeing* for a time, before venturing on the more trying exercise of *inspecting*. The sympathetic connexions which the eyes have with the other parts of the economy in a state of derangement, is a third cause mentioned by M. Pétrequin, and one which evidently points out the propriety of the strictest attention to the non-naturals.

There is nothing new in the *remedies* employed by M. Pétrequin. They consist chiefly in bleeding, generally and locally; locally, by leeches round the anus very frequently; purgatives; mercury; counter-irritation; and the endermic employment of strychnia. It is, therefore, to a selection of the proper season for administering the remedies, to a perseverance in their use, and to the having his patients constantly under his observation in an hospital, that the success of M. Pétrequin must be attributed. He ascribes much to the powers of strychnia, which he uses always externally, never internally. What he employs is prepared by M. Pelletier. M. Pétrequin applies it pure, in preference to mixing it up with fatty substances in the form of ointment. As it is applied in doses of a quarter to a third of a grain, a quantity from its smallness apt to be lost, he mixes it with a grain or two of powdered nux vomica. The blisters which precedes the application of the strychnia, M. Pétrequin forms instantaneously by means of a strong ammoniacal salve. This makes a clean, moist surface, on which to sprinkle the strychnia. At each application afterwards, care is taken to remove the false membrane which has formed in the interval. This, however, is insufficient to prevent the absorption diminishing as the wound dries, so that the doses may be increased, till a new vesication is deemed necessary.

An adjuvant of considerable value is the tincture of nux vomica, prepared with four ounces of the powder to a quart of brandy. This is used for stimulating the branches of the fifth nerve round the orbit, and thus acting sympathetically on the retina.

As strychnia is one of the remedies upon which M. Pétrequin chiefly depends in the treatment of amaurosis, it is important to observe, that it is only when the disease exists in the state of a pure asthenia, that he has recourse to it. The morbid elements which existed in combination with the asthenic state being removed by other means, this stimulant, as it is generally regarded, may be employed with hopes of advantage. M. Pétrequin regards it as an excitant both of the circulating and of the nervous system. It is a remedy, then, to be avoided in congestive or inflammatory cases. These require bleeding, purging, and mercury.

The notion generally entertained respecting nux vomica, and its alcaloid, strychnia, is, that they are powerfully stimulating. This belief is built, first, upon the admission that these substances cure palsies, which are presumed to be asthenic, and second, upon the fact that they give rise to tetanic spasms. Hence the rule, above referred to, that we are to abstain from using strychnia, where there are signs of plethora about the eye or the brain. But the school of Rasori maintain a totally different doctrine. M. Rognetta, for example, is of opinion that far from being an excitant, strychnia is a hyposthenisant or sedative, and that it depresses the powers of life, like bleeding or like belladonna. M. Rognetta also asserts that strychnia produces no effect, except by being taken into the circulation. He therefore gives it internally, in the form of an acetate; the dose varying from one twentieth of a grain to half a grain.

We have not hesitated to state very plainly what we consider M. Pétrequin's chief fault. We cannot quit him without noticing one particular which has given us great pleasure in the perusal of his work, namely, his energetic perseverance in endeavouring to accomplish the cure of his patients. We may, perhaps, be permitted to say that this, along with the reputation for success in treating cases of blindness, which he must have acquired, must have powerfully promoted the cure of such a disease as amaurosis—a nervous disorder, in many instances, of asthenic character, and, therefore, requiring the aid of a beneficial stimulus to the mind, such as that afforded by the exercise of hope, and the confident expectation of relief. We believe, that there is perhaps no disease in which moral influences of this kind are of so much importance as amaurosis; while, on the other hand, nothing proves so hurtful, as when the patient is either informed directly, or gathers from the dry, indifferent, inattentive, or repulsive manner of the practitioner, that the disease is hopeless.

---

